# ﻿Six new species of the orb-weaver spider genus *Araneus* Clerck, 1757 (Araneae, Araneidae) and a redescription of *A.colubrinus* Song & Zhu, 1992 from Fanjingshan National Nature Reserve, Guizhou, China

**DOI:** 10.3897/zookeys.1173.106315

**Published:** 2023-08-04

**Authors:** Xiaoqi Mi, Cheng Wang, Jiahui Gan

**Affiliations:** 1 College of Agriculture and Forestry Engineering and Planning, Guizhou Provincial Key Laboratory of Biodiversity Conservation and Utilization in the Fanjing Mountain Region, Tongren University, Tongren 554300, Guizhou, China Tongren University Tongren China

**Keywords:** Arachnida, morphology, new synonym, taxonomy, Wuling Mountains

## Abstract

Six new species of the genus *Araneus* Clerck, 1757 from Fanjingshan National Nature Reserve in Guizhou Province, China are described: *Araneuschenjingi***sp. nov.** (♂♀), and *A.yuboi***sp. nov.** (♂♀) are assigned to the *A.diadematus* group; *A.lihaiboi***sp. nov.** (♂♀), *A.shii***sp. nov.** (♂♀), *A.wanghuai***sp. nov.** (♂♀), and *A.yangchuandongi***sp. nov.** (♂♀) are assigned to the *A.sturmi* group. *Araneuscolubrinus* Song & Zhu, 1992 is redescribed. A new synonym of *Araneuscolubrinus* Song & Zhu, 1992 is proposed: *Araneusoctodentalis* Song & Zhu, 1992 **syn. nov.**

## ﻿Introduction

Fanjingshan National Nature Reserve lies in northeast Guizhou Province, southwest China, the main peak of Wuling Mountains, with the highest altitude of 2572 m. It is the only habitat of Guizhou snub-nosed monkey (*Rhinopithecusbrelichi* Thomas). The spider fauna of this region was first deeply investigated by Song et al. (2006), who recorded a total of 126 species in 18 families. The spider fauna of this region has increased in recent years (Zhang et al. 2009; Huang et al. 2015; Lu et al. 2015; Wang et al. 2015, 2016, 2018, 2020a, b, c; Zeng et al. 2016; Jiang et al. 2018; Li et al. 2019; Wang and Wang 2020; Li et al. 2021, 2022; Yang et al. 2022). At present, 386 species in 34 families have been found in this region (pers. obs.).

*Araneus* Clerck, 1757 is the largest genus of the orb-weaver spider family Araneidae, and the Chinese *Araneus* fauna was summarized by Wu et al. (2023), with a total of 121 *Araneus* species recorded in China at present (WSC 2023). Specimens of *Araneus* collected in Fanjingshan National Nature Reserve were identified, a total of 24 species was recognized, including six new species. The goal of this paper is to describe the new species and redescribe *Araneuscolubrinus* Song & Zhu, 1992.

## ﻿Material and method

All the specimens were collected by beating shrubs or hand collecting and are preserved in 75% ethanol. Type specimens of the new species are deposited in the Museum of Tongren University, China (**TRU**). The specimens were examined with an Olympus SZX16 stereomicroscope. The epigynes were cleared in lactic acid for examination and imaging. The left male pedipalp was dissected in ethanol for examination, description, and imaging, and was expanded in lactic acid when necessary. Photographs of the habitus and copulatory organs were taken with a Kuy Nice digital camera mounted on an Olympus BX43 compound microscope. Compound focus images were generated using Helicon Focus v. 6.7.1. The paths of the left copulatory ducts were drawn using Adobe Illustrator CC 2018.

All measurements are given in millimeters. Leg measurements are given as total length (femur, patella + tibia, metatarsus, tarsus). Abbreviations used in the text and figures are as follows: **ALE** anterior lateral eye; **AME** anterior median eye; **C** conductor; **CD** copulatory duct; **CO** copulatory opening; **E** embolus; **EB** embolic base; **EL** embolic lamella; **EN** embolic node; **ET** embolic tooth; **FD** fertilization duct; **MA** median apophysis; **MOA** median ocular area; **PLE** posterior lateral eye; **PME** posterior median eye; **Sc** scape; **Sp** spermatheca; **ST** subterminal apophysis; **TA** terminal apophysis.

## ﻿Taxonomic account

### ﻿Family Araneidae Clerck, 1757

#### ﻿Genus Araneus

Taxon classificationAnimaliaAraneaeAraneidae

Clerck, 1757

CE459ADF-B438-5DD2-9A91-CB35956F0901

Araneus Clerck, 1757: 22

##### Type species.

*Araneusangulatus* Clerck, 1757.

##### Comment.

We place the six new species in the genus *Araneus* provisionally because they share very similar habitus and copulatory organs with other *Araneus* species, although they are very different from the generotype *Araneusangulatus* in somatic and genitalic structures. Grouping of the species is according to Yin et al. (1997).

#### ﻿ Araneus chenjingi sp. nov.

Taxon classificationAnimaliaAraneaeAraneidae

38B08EB1-6194-5902-A8E3-67EC98AA0AC7

https://zoobank.org/A395AE89-B28B-4CCB-A80C-DC4D853081C0

**Figure 1.**
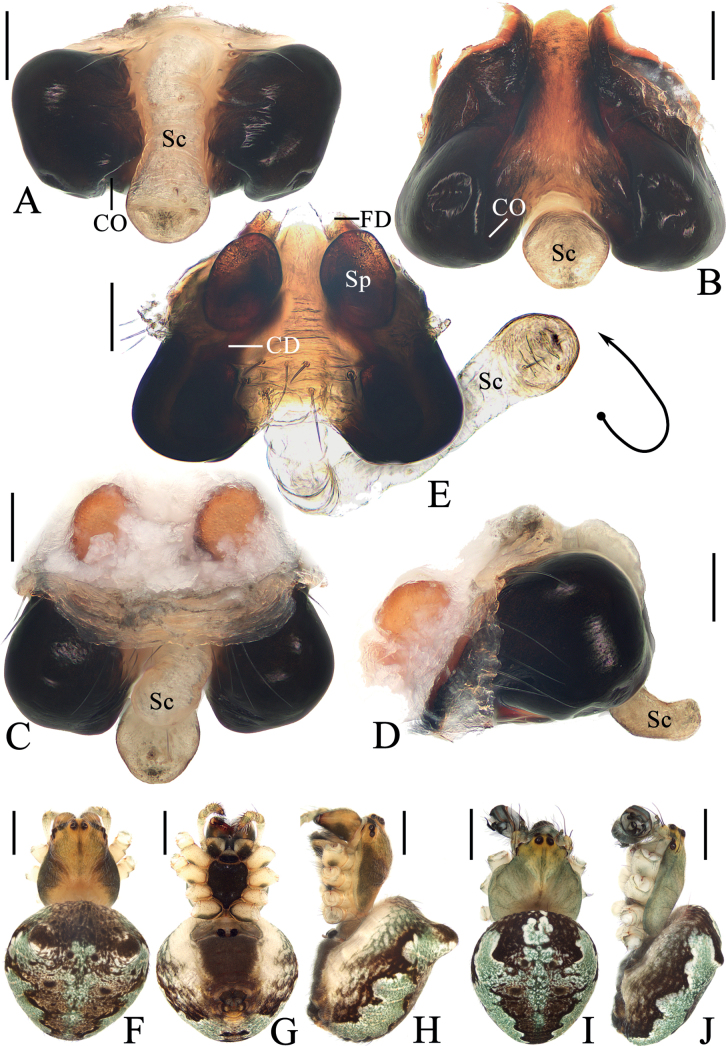
*Araneuschenjingi* sp. nov. **A–H** female paratype TRU-Araneidae-179 **I, J** male holotype **A** epigyne, ventral view **B** ibid., posterior view **C** ibid., anterior view **D** ibid., lateral view **E** vulva, anterior view **F** habitus, dorsal view **G** ibid., ventral view **H** ibid., lateral view **I** ibid., dorsal view **J** ibid., lateral view. Scale bars: 0.1 mm (**A–E**); 1 mm (**F–J**). Abbreviations: CD copulatory duct, CO copulatory opening, FD fertilization duct, Sc scape, Sp spermatheca.

**Figure 2.**
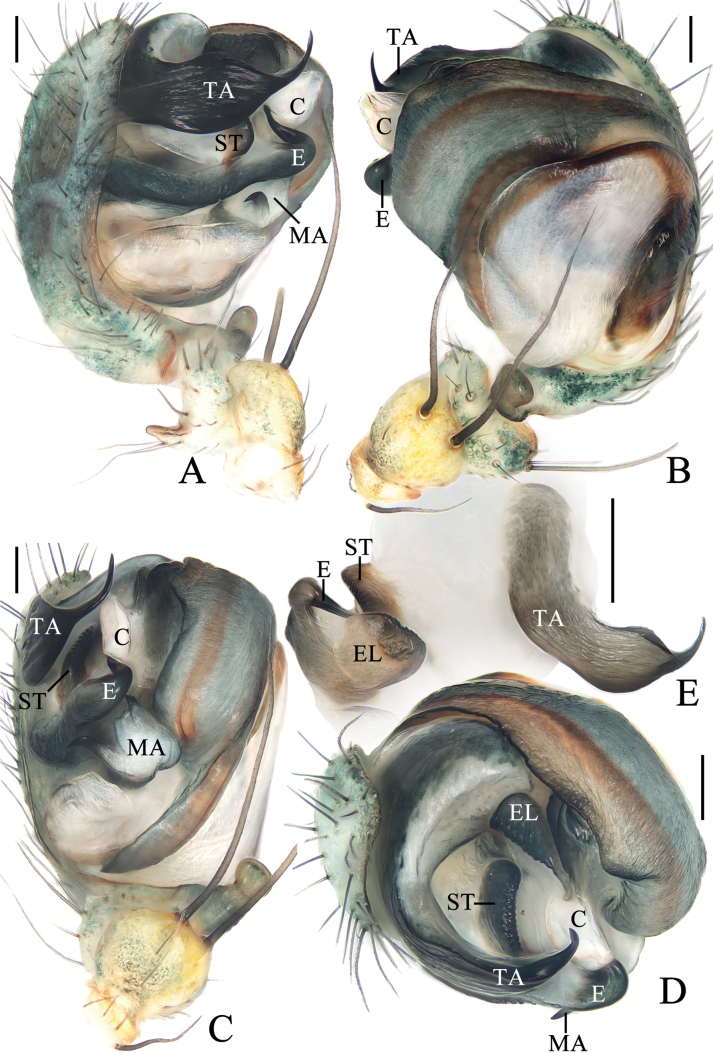
*Araneuschenjingi* sp. nov. male holotype **A** pedipalp, prolateral view **B** ibid., retrolateral view **C** ibid., ventral view **D** ibid., apical view **E** part of expanded bulb. Scale bars: 0.1 mm. Abbreviations: C conductor, E embolus, EL embolic lamella, MA median apophysis, ST subterminal apophysis, TA terminal apophysis.

Figs 1 , 2 , 15A–D , 17

##### Type material.

***Holotype*** ♂ (TRU-Araneidae-178), China: Guizhou Province, Tongren City, Songtao Miao Autonomous County, Wuluo Township, Taohuayuan Village, Yangaoping (27°58.71'N, 108°45.86'E, ca 1620 m), 2.IV.2022, X.Q. Mi et al. leg. ***Paratypes***: 3♀ (TRU-Araneidae-179–181), same data as holotype.

##### Diagnosis.

The new species resembles *A.seminiger* (L. Koch, 1878) in coloration of abdomen, but differs in: 1) conductor membranous, without pointed tip (Fig. 2A–D) vs heavily sclerotized with a pointed tip (Tanikawa 2007: fig. 644); 2) embolus visible in prolateral view (Fig. 2A) vs completely hidden (Tanikawa 2007: fig. 644); 3) median apophysis about equal width to length in prolateral view (Fig. 2A) vs ~ 2× longer than wide (Tanikawa 2007: fig. 644); and 4) scape short, distal end slightly beyond epigastric furrow (Fig. 1A) vs long, far exceeding epigastric furrow (Tanikawa 2007: fig. 643).

##### Description.

**Male** (holotype, Figs 1I, J, 2, 15A–D). Total length 4.10. Carapace 2.15 long, 1.85 wide. Abdomen 2.85 long, 2.45 wide. Clypeus 0.03 high. Eye sizes and interdistances: AME 0.09, ALE 0.08, PME 0.10, PLE 0.08, AME–AME 0.18, AME–ALE 0.28, PME–PME 0.15, PME–PLE 0.40, MOA length 0.33, anterior width 0.35, posterior width 0.33. Leg measurements: I 7.95 (2.35, 2.85, 2.00, 0.75), II 6.70 (2.00, 2.35, 1.65, 0.70), III 4.35 (1.50, 1.40, 0.95, 0.50), IV 5.90 (1.95, 1.95, 1.45, 0.55). Carapace pear-shaped, yellow to pale green, cervical groove obvious, fovea depressed. Chelicerae yellow, four promarginal teeth and three retromarginal teeth. Endites almost square, with tooth-like process laterally, labium triangular, both yellow to pale green. Sternum cordiform, yellow to pale green, with dark setae. Legs yellow to pale green with dark green annuli, tibia I with 15 macrosetae, tibia II with 13 macrosetae, tibia III with eight macrosetae, tibia IV with 11 macrosetae. Abdomen oval, ~ 1.15× longer than wide, with a pair of very low humps, dorsum pale green with a whitish green spot surrounding by dark markings anteriorly and a triangular dark patch posteriorly; venter grayish brown. Spinnerets yellowish green.

***Pedipalp*** (Fig. 2): with a basal femoral protrusion; patella with two bristles; median apophysis about equal length to width, with two-pointed tip on opposite sides; embolus slender, almost transversal in prolateral view, curved sharply distally; conductor membranous, widest at base in ventral view; terminal apophysis wide at base, pointed distally, curved into a C-shape in apical view; subterminal apophysis blunt at tip.

**Female** (paratype TRU-Araneidae-179, Fig. 1A–H). Total length 5.15. Carapace 2.25 long, 1.90 wide. Abdomen 3.45 long, 3.25 wide. Clypeus 0.05 high. Eye sizes and interdistances: AME 0.10, ALE 0.09, PME 0.11, PLE 0.09, AME–AME 0.18, AME–ALE 0.38, PME–PME 0.15, PME–PLE 0.50, MOA length 0.35, anterior width 0.38, posterior width 0.38. Leg measurements: I 7.60 (2.45, 2.70, 1.70, 0.75), II 6.70 (2.15, 2.35, 1.50, 0.70), III 4.00 (1.30, 1.30, 0.85, 0.55), IV 5.95 (1.95, 2.05, 1.35, 0.60). Habitus similar to that of male but the humps on dorsal abdomen a bit higher, and carapace yellow with dark thoracic bilateral sub-margin, endites, labium, sternum, and spinnerets much darker.

***Epigyne*** (Fig. 1A–E): base of epigyne heavily sclerotized, scape with nearly parallel sides, distal end spoon shaped; copulatory openings arcuated, on the posterior surface; copulatory ducts longer than the spermatheca, twisted into a C-shape; spermathecae oval, about half the spermatheca width apart.

##### Variation.

Total length: ♀♀ 4.15–5.20 (*n* = 3).

##### Distribution.

Known only from type locality.

##### Comments.

The wide oval female abdomen with a pair of anterior lateral humps, and the long, ridged scape indicate that the new species belongs to the *A.diadenmatus* group.

##### Etymology.

The species is named after Mr. Jing Chen (Fanjingshan National Nature Reserve Administration Bureau), who offered help with specimen collection for this research; noun in genitive case.

#### ﻿ Araneus colubrinus

Taxon classificationAnimaliaAraneaeAraneidae

Song & Zhu, 1992

1A0F90CA-DC14-5024-B2D0-9801A573846A

**Figure 3.**
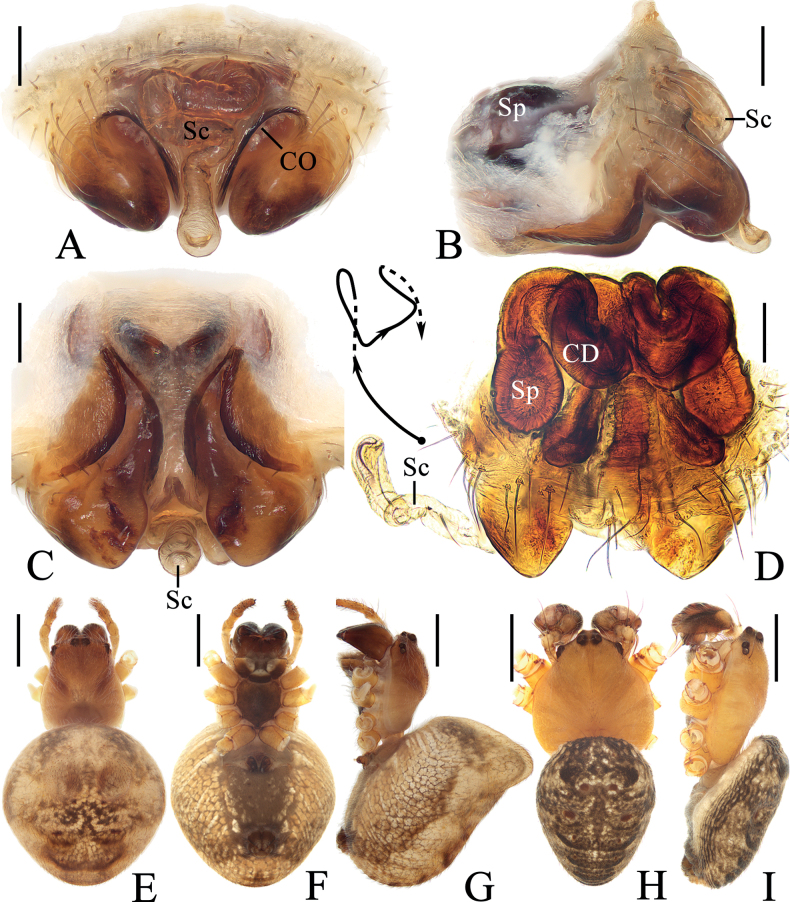
*Araneuscolubrinus* Song & Zhu, 1992 **A–G** female TRU-Araneidae-183 **H, I** male TRU-Araneidae-182 **A** epigyne, ventral view **B** ibid., lateral view **C** ibid., posterior view **D** vulva, anterior view **E** habitus, dorsal view **F** ibid., ventral view **G** ibid., lateral view **H** ibid., dorsal view **I** ibid., lateral view. Scale bars: 0.1 mm (**A–D**); 1 mm (**E–I**). Abbreviations: CD copulatory duct, CO copulatory opening, Sc scape, Sp spermatheca.

**Figure 4.**
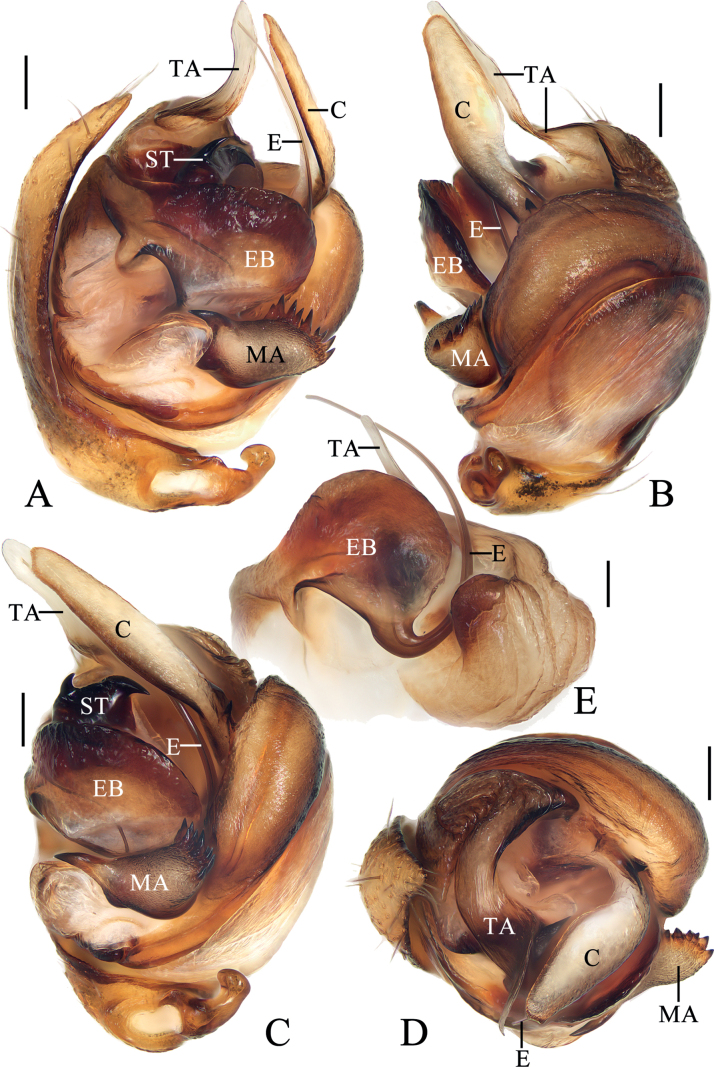
*Araneuscolubrinus* Song & Zhu, 1992, male TRU-Araneidae-182 **A** pedipalp, prolateral view **B** ibid., retrolateral view **C** ibid., ventral view **D** ibid., apical view **E** part of expanded bulb. Scale bars: 0.1 mm. Abbreviations: C conductor, E embolus, EB embolic base, MA median apophysis, ST subterminal apophysis, TA terminal apophysis.

Figs 3 , 4 , 15E–H , 17

Araneus colubrinus Song & Zhu, 1992: 169, fig. 4A, B; Song and Li 1997: 413, fig. 16A, B; Yin et al. 1997: 146, fig. 61a–c; Song et al. 1999: 238, fig. 136S, T (type material not examined).
Araneus octodentalis Song & Zhu, 1992: 169, fig. 5A, B; Song and Li 1997: 414, fig. 17A, B; Yin et al. 1997: 149, fig. 64a–c; Song et al. 1999: 240, fig. 144A, B (syn. nov., type material not examined).

##### Material examined.

4♂5♀ (TRU-Araneidae-182–190), China: Guizhou Province, Tongren City, Yinjiang Tujia and Miao Autonomous County, Ziwei Township, Dayuanzhi Village, Huguosi (27°54.54'N, 108°46.57'E, ca 1660 m), 9.V.2020, X.Q. Mi et al. leg.

##### Diagnosis.

**Figure 5.**
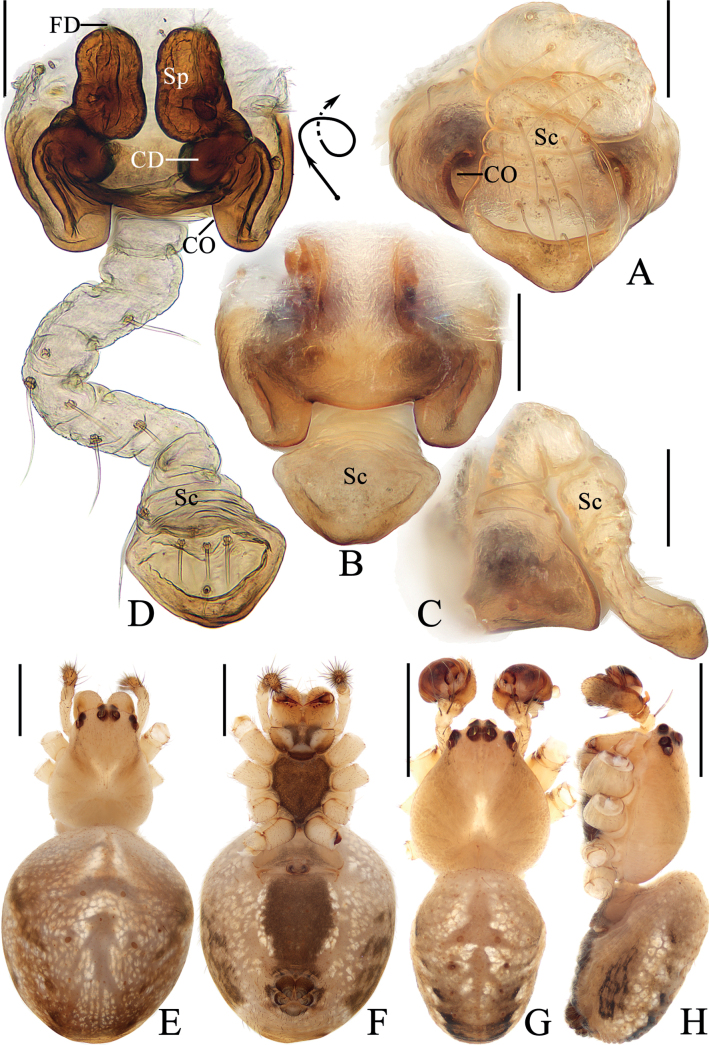
*Araneuslihaiboi* sp. nov. **A–F** female paratype TRU-Araneidae-192 **G, H** male holotype **A** epigyne, ventral view **B** ibid., posterior view **C** ibid., lateral view **D** vulva, posterior view **E** habitus, dorsal view **F** ibid., ventral view **G** ibid., dorsal view **H** ibid., lateral view. Scale bars: 0.1 mm (**A–D**); 1 mm (**E–H**). Abbreviations: CD copulatory duct, CO copulatory opening, FD fertilization duct, Sc scape, Sp spermatheca.

**Figure 6.**
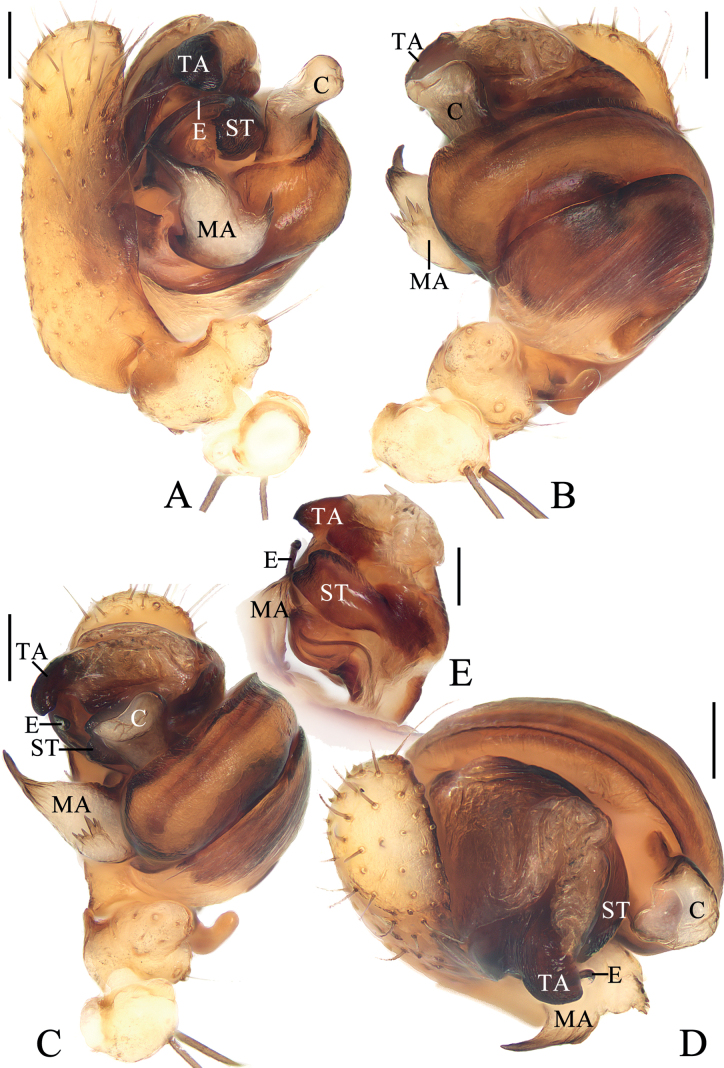
*Araneuslihaiboi* sp. nov. male holotype **A** pedipalp, prolateral view **B** ibid., retrolateral view **C** ibid., ventral view **D** ibid., apical view **E** part of expanded bulb. Scale bars: 0.1 mm. Abbreviations: C conductor, E embolus, MA median apophysis, ST subterminal apophysis, TA terminal apophysis.

This species resembles *A.yangchuandongi* sp. nov., *A.conexus* Liu, Irfan, Yang & Peng, 2019, and *A.zhoui* Mi & Wang, 2023 in somatic morphology, but it can be distinguished from *A.yangchuandongi* sp. nov. in: 1) carapace lacking macrosetae anterior to fovea (Fig. 3E, H) vs having ten macrosetae (Fig. 11E, G); 2) epigyne scape twisted into an S-shape (Fig. 3A) vs almost straight (Fig. 11A, B); 3) spermathecae oval (Fig. 3D) vs spherical (Fig. 11D); 4) spermathecae separated by ~ 1.6× of the spermathecae width (Fig. 3D) vs nearly touching each other (Fig. 11D); 5) terminal apophysis membranous (Fig. 4A–E) vs pointed and heavily sclerotized (Fig. 12A–E); 6) conductor ~ 3.6× longer than wide in retrolateral view (Fig. 4B) vs about equal length to width (Fig. 12B); and 7) embolus threadlike (Fig. 4A–E) vs tapered (Fig. 12C, D). It differs *A.conexus* in: 1) the epigyne scape extremely twisted into an S-shape (Fig. 3A) vs almost straight (Liu et al. 2019: fig. 4A, B); 2) spermathecae separated by ~ 1.6× of the spermathecae width (Fig. 3D) vs nearly touching each other (Liu et al. 2019: fig. 4C, D); 3) terminal apophysis membranous (Fig. 4A–E) vs pointed (Liu et al. 2019: figs 2A, B, D, E, 3, 5C); 4) subterminal apophysis with two spurs (Fig. 4A, C) vs lacking spurs (Liu et al. 2019: figs 2A, D, 3A–D, 5C); 5) embolus threadlike (Fig. 4A–E) vs tapered (Liu et al. 2019: figs 2A, D, 3A–D, 5C); and 6) conductor ~3.6× longer than wide in retrolateral view (Fig. 4B) vs slightly wider than long (Liu et al. 2019: figs 2A, B, D, E, 3A, B, 5C). It differs from *A.zhoui* in: 1) female carapace lacking short spines anterior to fovea (Fig. 3E, H) vs with two short spines (Wu et al. 2023: fig. 7H); 2) epigyne scape extremely twisted into an S-shape (Fig. 3A) vs slightly twisted (Wu et al. 2023: fig. 7A, B); 3) spermathecae separated by ~ 1.6× of the spermathecae width (Fig. 3D) vs nearly touching each other (Wu et al. 2023: fig. 7D); 4) conductor ~ 3.6× longer than wide in retrolateral view (Fig. 4B) vs slightly wider than long (Wu et al. 2023: fig. 8C); 5) embolus threadlike (Fig. 4A–E) vs tapered (Wu et al. 2023: fig. 8A, E); and 6) subterminal apophysis having two spurs (Fig. 4A, C) vs lacking (Wu et al. 2023: fig. 8A, E).

##### Description.

**Male** (TRU-Araneidae-182, Figs 3H, I, 4, 15E–H). Total length 3.85. Carapace 2.05 long, 1.80 wide. Abdomen 2.40 long, 1.75 wide. Clypeus 0.08 high. Eye sizes and interdistances: AME 0.09, ALE 0.08, PME 0.10, PLE 0.08, AME–AME 0.18, AME–ALE 0.25, PME–PME 0.15, PME–PLE 0.38, MOA length 0.30, anterior width 0.30, posterior width 0.35. Leg measurements: I 7.35 (2.20, 2.50, 1.90, 0.75), II 6.45 (1.95, 2.20, 1.60, 0.70), III 3.85 (1.30, 1.25, 0.80, 0.50), IV 5.55 (1.80, 1.80, 1.35, 0.60). Carapace pear-shaped, yellowish brown with dark setae, cervical groove slightly obvious, fovea depressed. Chelicerae yellow, four promarginal teeth and three retromarginal teeth. Endites square, yellow, with tooth-like process laterally, labium triangular, grayish yellow, both with pale tip. Sternum cordiform, grayish yellow with dark setae. Legs yellow to yellowish brown, without annuli, tibia I with 13 macrosetae, distally with constriction (see arrow in Fig. 15E), tibia II with 12 macrosetae, tibia III with nine macrosetae, tibia IV with 11 macrosetae. Abdomen oval, blunt anteriorly, ~ 1.37× longer than wide, covered with dense setae, dorsum grayish yellow with three pairs of dark lateral patches posteriorly; venter grayish yellow with pair of longitudinal yellow patches laterally. Spinnerets yellowish brown.

***Pedipalp*** (Fig. 4): with basal femoral protrusion; patella with two bristles; median apophysis large, with a pointed tip and ten teeth; embolus slender, longer than conductor; conductor membranous ~ 3.6× longer than wide in retrolateral view, with a spur at base; terminal apophysis membranous, approximately equal in length to conductor; subterminal apophysis heavily sclerotized, with two spurs.

**Female** (TRU-Araneidae-183, Fig. 3A–G). Total length 4.75. Carapace 1.90 long, 1.60 wide. Abdomen 3.75 long, 3.20 wide. Clypeus 0.05 high. Eye sizes and interdistances: AME 0.09, ALE 0.08, PME 0.11, PLE 0.08, AME–AME 0.15, AME–ALE 0.10, PME–PME 0.15, PME–PLE 0.38, MOA length 0.30, anterior width 0.28, posterior width 0.35. Leg measurements: I 6.25 (1.95, 2.25, 1.40, 0.65), II 5.45 (1.70, 1.95, 1.20, 0.60), III 3.50 (1.20, 1.10, 0.70, 0.50), IV 5.00 (1.60, 1.75, 1.10, 0.55). Habitus similar to that of male but anterior abdominal elevation more obvious; chelicerae, endites, labium, and sternum darker, and abdominal patches much paler.

***Epigyne*** (Fig. 3A–D): ~ 1.69× wider than long at base; scape twisted into an S-shape, distally spoon shaped; copulatory openings strongly concave, on ventral surface; copulatory ducts extremely long, middle part twisted into a U-shape; spermathecae oval, ~ 1.6× of the spermathecae width apart.

##### Variation.

Total length: ♂♂ 3.65–3.85 (*n* = 4); ♀♀ 4.65–5.20 (*n* = 5).

##### Distribution.

Hubei, Guizhou.

##### Justification of the synonymy.

The holotypes of *Araneuscolubrinus* (only known from a single female) and *Araneusoctodentalis* (only known from a single male) were not examined, but both species can be easily recognized due to the perfect illustrations (Song and Zhu 1992). Although the anterior abdominal elevation of female more obvious than that of male, but both sexes have the same color pattern on dorsal abdomen, the same dimorphism that also exists in some related species, such as *A.conexus*, *A.zhoui*, and *A.yangchuandongi* sp. nov. Moreover, both the holotypes of *A.colubrinus* and *A.octodentalis* were collected on the same day in Wuling Mountains (Badong County, Hubei Province). Hence we propose that *Araneuscolubrinus* Song & Zhu, 1992 is a senior synonym of *Araneusoctodentalis* Song & Zhu, 1992.

#### ﻿ Araneus lihaiboi sp. nov.

Taxon classificationAnimaliaAraneaeAraneidae

0E6DB518-AA4A-5F41-BD46-0F8F3542A411

https://zoobank.org/97402F68-706F-4F48-B8AB-BD0AF42BD4FE

Figs 5 , 6 , 15I–J , 17

##### Type material.

***Holotype*** ♂ (TRU-Araneidae-191), China: Guizhou Province, Tongren City, Yinjiang Tujia and Miao Autonomous County, Ziwei Township, Dayuanzhi Village, Mianxüling (27°54.89'N, 108°40.17'E, ca 1690 m), 14.VI.2020, C. Wang & J.H. Gan leg. ***Paratypes***: 1♂3♀ (TRU-Araneidae-192–195), same data as for holotype; 1♂ (TRU-Araneidae-196), Huguosi (27°54.72'N, 108°28.62'E, ca 1500 m), 25.IV.2020, X.Q. Mi & C. Wang leg.

##### Diagnosis.

The new species resembles *A.falcatus* Guo, Zhang & Zhu, 2011 in somatic and genital structures, but differs in: 1) embolus slightly curved in prolateral view (Fig. 6A) vs strongly curved into a C-shape (Guo et al. 2011: fig. 4); 2) embolus close to terminal apophysis (Fig. 6A) vs widely seperated (Guo et al. 2011: fig. 4); 3) subterminal apophysis present (Fig. 6A, C–E) vs absent (Guo et al. 2011: fig. 4); 4) female abdomen lacking anterolateral white bands (Fig. 5A) vs present (Guo et al. 2011: fig. 1); and 5) chelicerae with four promarginal teeth vs three promarginal teeth.

##### Description.

**Male** (holotype, Figs 5G, H, 6, 15I–L). Total length 2.90. Carapace 1.45 long, 1.25 wide. Abdomen 1.70 long, 1.23 wide. Clypeus 0.05 high. Eye sizes and interdistances: AME 0.08, ALE 0.08, PME 0.10, PLE 0.08, AME–AME 0.09, AME–ALE 0.11, PME–PME 0.06, PME–PLE 0.18, MOA length 0.23, anterior width 0.23, posterior width 0.25. Leg measurements: I 5.15 (1.55, 1.80, 1.20, 0.60), II 4.65 (1.40, 1.65, 1.05, 0.55), III 2.95 (1.00, 0.95, 0.60, 0.40), IV 3.90 (1.25, 1.30, 0.90, 0.45). Carapace pear-shaped, yellow with pale, longitudinal patch anterior to fovea, cervical groove slightly distinct. Chelicerae yellow, four promarginal teeth and three retromarginal teeth. Endites square, yellowish brown, with tooth-like process laterally, labium triangular, brown with paler tip. Sternum cordiform, yellowish brown with pale setae. Legs yellow without annulus, tibia I with ten macrosetae, tibia II with ten macrosetae, tibia III with six macrosetae, and tibia IV with eight macrosetae. Abdomen oval, ~ 1.4× longer than wide, covered with pale setae, dorsum yellow to scaly white, with five pairs of lateral dark patches; venter grayish brown. Spinnerets brown.

***Pedipalp*** (Fig. 6): with basal femoral protrusion; patella with two bristles; median apophysis large, bifurcated, dorsal ramus long, curved, pointed at tip, ventral ramus short with serrated tip; embolus slender, slightly anti-clockwise curved in prolateral view; conductor weakly sclerotized, wider at tip; terminal apophysis arched, heavily sclerotized distally; subterminal apophysis heavily sclerotized, somewhat rounded in prolateral view.

**Female** (paratype TRU-Araneidae-192, Fig. 5A–F). Total length 4.70. Carapace 1.95 long, 1.45 wide. Abdomen 3.20 long, 2.65 wide. Clypeus 0.05 high. Eye sizes and interdistances: AME 0.09, ALE 0.09, PME 0.11, PLE 0.09, AME–AME 0.10, AME–ALE 0.20, PME–PME 0.10, PME–PLE 0.25, MOA length 0.28, anterior width 0.28, posterior width 0.30. Leg measurements: I 5.65 (1.65, 2.10, 1.30, 0.60), II 5.00 (1.50, 1.80, 1.15, 0.55), III 3.10 (1.00, 1.05, 0.65, 0.40), IV 4.60 (1.45, 1.65, 1.00, 0.50). Habitus similar to that of male but cervical groove more distinct.

***Epigyne*** (Fig. 5A–D): ~ 1.4× wider than long; scape twisted into an S-shape; copulatory openings strongly concave, located at lateral sides of ventral surface; copulatory ducts coiled 360° medially; spermathecae kidney-shaped, less than 1/4 the spermatheca width apart.

##### Variation.

Total length: ♂♂ 2.60–2.90 (*n* = 3); ♀♀ 3.75–4.70 (*n* = 3).

##### Distribution.

Known only from type locality.

##### Comments.

The oval abdomen, the long, twisted, and ridged scape, and the male pedipalp with a bifurcated median apophysis and arched terminal apophysis indicate that the new species belongs to the *A.sturmi* group.

##### Etymology.

The species is named after Mr. Haibo Li (Fanjingshan National Nature Reserve Administration Bureau), who provided tremendous support during fieldwork; noun in genitive case.

#### ﻿ Araneus shii sp. nov.

Taxon classificationAnimaliaAraneaeAraneidae

582F7681-3929-5071-BA38-7E52C1CD2C96

https://zoobank.org/2B5FF64C-C28F-40F5-A854-61983F2AFFAB

**Figure 7.**
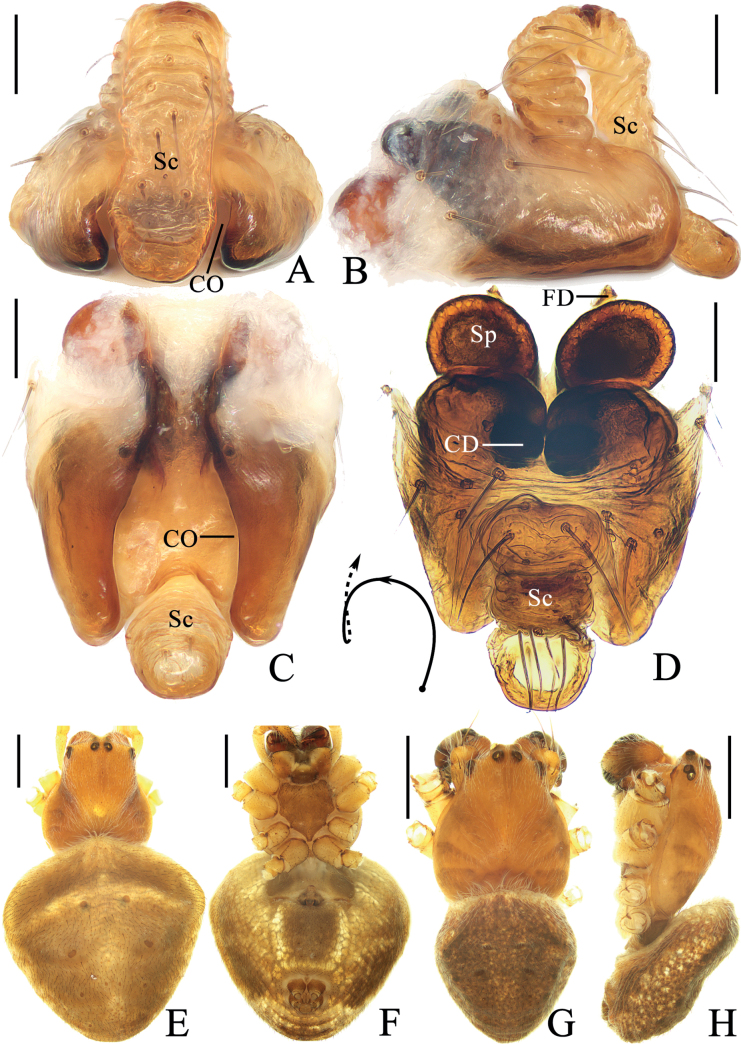
*Araneusshii* sp. nov. **A–F** female paratype TRU-Araneidae-198 **G, H** male holotype **A** epigyne, ventral view **B** ibid., lateral view **C** ibid., posterior view **D** vulva, anterior view **E** habitus, dorsal view **F** ibid., ventral view **G** ibid., dorsal view **H** ibid., lateral view. Scale bars: 0.1 mm (**A–D**); 1 mm (**E–H**). Abbreviations: CD copulatory duct, CO copulatory opening, FD fertilization duct, Sc scape, Sp spermatheca.

**Figure 8.**
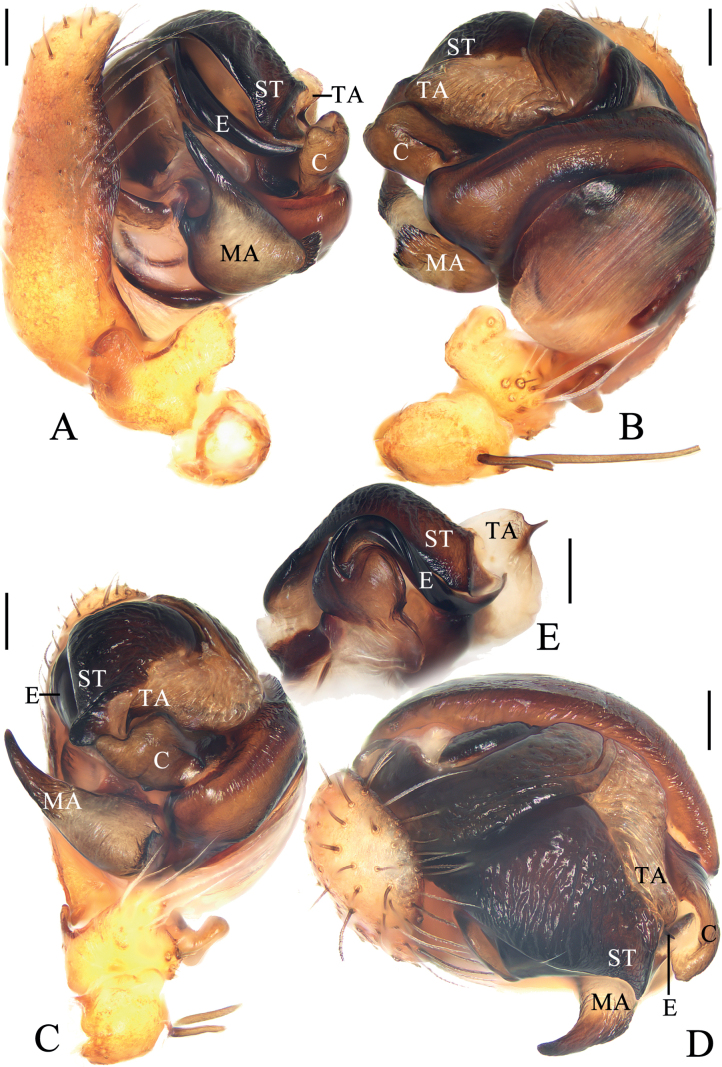
*Araneusshii* sp. nov. male holotype **A** pedipalp, prolateral view **B** ibid., retrolateral view **C** ibid., ventral view **D** ibid., apical view **E** part of expanded bulb. Scale bars: 0.1 mm. Abbreviations: C conductor, E embolus, MA median apophysis, ST subterminal apophysis, TA terminal apophysis.

Figs 7 , 8 , 15M–P , 17

##### Type material.

***Holotype*** ♂ (TRU-Araneidae-197), China: Guizhou Province, Tongren City, Yinjiang Tujia and Miao Autonomous County, Muhuang Township, Jinchang Village, Maxi’ao (28°1.37'N, 108°45.00'E, ca 1340 m), 10.V.2020, X.Q. Mi et al. leg. ***Paratypes***: 4♀ (TRU-Araneidae-198–201), same data as for holotype; 3♂6♀ (TRU-Araneidae-202–210), same locality, 26.IV.2020, X.Q. Mi & C. Wang leg.; 1♀ (TRU-Araneidae-211), Ziwei Township, Dayuanzhi Village, Huguosi (27°54.54'N, 108°46.57'E, ca 1660 m), 9.V.2020, X.Q. Mi et al. leg.; 1♀ (TRU-Araneidae-212), Mianxüling (27°54.89'N, 108°40.17'E, ca 1790 m), 23.VII.2021, X.Q. Mi et al. leg.

##### Diagnosis.

Females of the new species resembles *A.flagelliformis* Zhu & Yin, 1998 in appearance, but can be distinguished as follows: 1) scape with nearly parallel sides in ventral view (Fig. 7A) vs twisted in an S-shape (Yin et al. 1997: fig. 84c); 2) scape slightly beyond epigastric furrow in ventral view (Fig. 7A) vs far exceeding epigastric furrow (Yin et al. 1997: fig. 84c); 3) anterolateral humps on dorsal abdomen very low (Fig. 7E) vs humps obvious (Yin et al. 1997: fig. 84a); and 4) total length less than 6 vs total length longer than 16. Males of the new species can be distinguished from congeneric species by the following combination of characters: 1) terminal apophysis weakly sclerotized with a fine tip (Fig. 8A, C, E); 2) subterminal apophysis heavily sclerotized, arched (Fig. 8A–E); 3) median apophysis large, bifurcated, dorsal ramus long, curved, pointed at tip, ventral ramus very short with serrated tip (Fig. 8A–D); 4) rather long embolus with a wide base (Fig. 8A, C, E); and 5) tibia I slightly curved (Fig. 15M).

##### Description.

**Male** (holotype, Figs 7G, H, 8, 15M–P). Total length 3.60. Carapace 2.10 long, 1.70 wide. Abdomen 2.20 long, 1.75 wide. Clypeus 0.08 high. Eye sizes and interdistances: AME 0.10, ALE 0.08, PME 0.13, PLE 0.08, AME–AME 0.18, AME–ALE 0.15, PME–PME 0.13, PME–PLE 0.33, MOA length 0.35, anterior width 0.38, posterior width 0.38. Leg measurements: I 6.70 (2.05, 2.55, 1.45, 0.65), II 5.80 (1.85, 2.20, 1.30, 0.45), III 3.70 (1.30, 1.25, 0.75, 0.40), IV 5.05 (1.65, 1.75, 1.15, 0.50). Carapace pear-shaped, yellow with inconspicuous grayish brown radial patches in thoracic region, with pale setae. Chelicerae yellow, four promarginal and three retromarginal teeth. Endites somewhat square, yellow with pale tip, with tooth-like process laterally, labium triangular, grayish yellow with paler tip. Sternum cordiform, yellow with pale and grayish brown setae. Legs yellow with yellowish brown annuli, tibia I slightly curved, with nine macrosetae, tibia II with 12 macrosetae, tibia III with seven macrosetae, and tibia IV with nine macrosetae. Abdomen wide oval, ~ 1.25× longer than wide, dorsum yellow to grayish brown, with dense, long, pale setae anteriorly and short, dark setae posteriorly; venter grayish brown with a pair of longitudinal white patches laterally. Spinnerets yellowish brown.

***Pedipalp*** (Fig. 8): with basal femoral protrusion; patella with two bristles; median apophysis large, bifurcated, dorsal ramus long, pointed at tip, ventral ramus very short with serrated tip; embolus prominent, slightly curved at tip in prolateral view; conductor somewhat square, widest at base in ventral view; terminal apophysis weakly sclerotized, with a pointed tip; subterminal apophysis heavily sclerotized, arched and covering most part of the embolus.

**Female** (paratype TRU-Araneidae-198, Fig. 7A–F). Total length 5.25. Carapace 2.20 long, 1.90 wide. Abdomen 3.60 long, 3.45 wide. Clypeus 0.08 high. Eye sizes and interdistances: AME 0.10, ALE 0.08, PME 0.13, PLE 0.08, AME–AME 0.15, AME–ALE 0.35, PME–PME 0.13, PME–PLE 0.43, MOA length 0.35, anterior width 0.38, posterior width 0.38. Leg measurements: I 5.70 (1.85, 2.00, 1.25, 0.60), II 5.20 (1.60, 1.90, 1.10, 0.60), III 3.30 (1.10, 1.10, 0.65, 0.45), IV 4.85 (1.60, 1.70, 1.05, 0.50). Habitus similar to that of male but abdomen a little paler.

***Epigyne*** (Fig. 7A–D): with a long base; scape long, extending forward first, then reflected to posterior, with nearly parallel sides and spoon shaped distal tip; copulatory openings wide and deeply concaved, at posterior surface; copulatory ducts curved about 90°; spermathecae elliptical, less than 1/4 of the spermatheca width apart.

##### Variation.

Total length: ♂♂ 3.45–3.80 (*n* = 4), ♀♀ 4.45–5.25 (*n* = 12).

##### Distribution.

Known only from type locality.

##### Comments.

The wide oval abdomen, long and ridged scape, and the male pedipalp with a bifurcated median apophysis indicate that the new species belongs to the *A.sturmi* group.

##### Etymology.

The specific name is a patronym of Mr. Lei Shi (Fanjingshan National Nature Reserve Administration Bureau), who helped us greatly with specimen collection on this research; noun in genitive case.

#### ﻿ Araneus wanghuai sp. nov.

Taxon classificationAnimaliaAraneaeAraneidae

06DA8CE9-13A0-5CD3-9170-BC18B2469FF4

https://zoobank.org/B342DB2C-AA82-4955-A90B-F24147357F40

**Figure 9.**
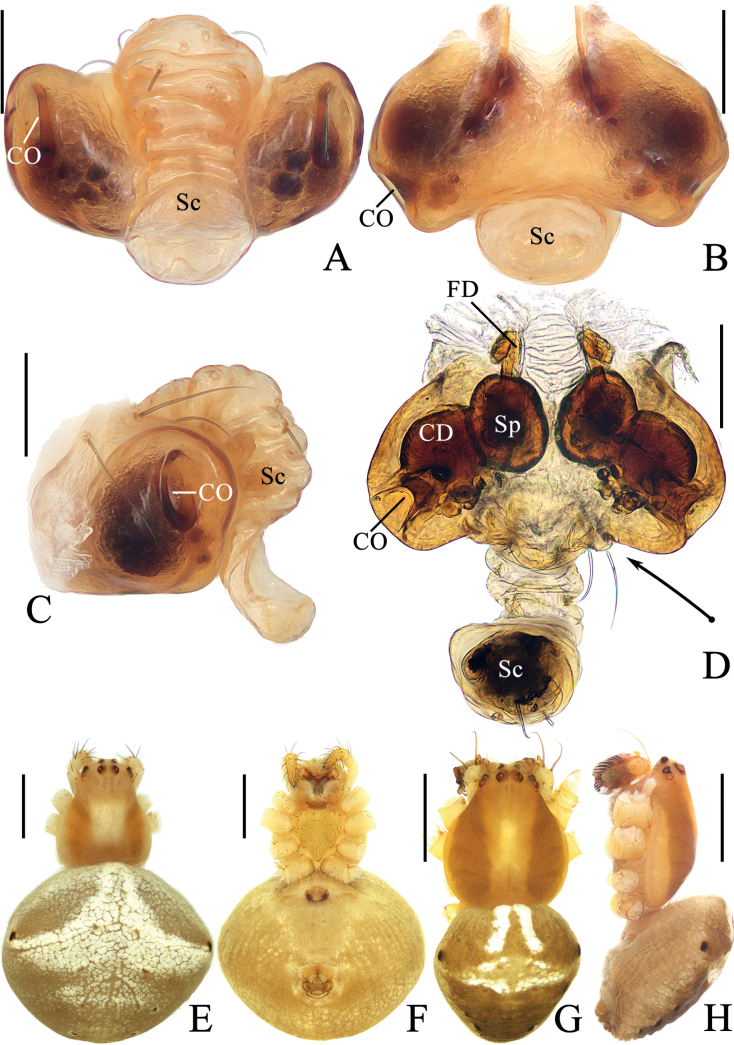
*Araneuswanghuai* sp. nov. **A–F** female paratype TRU-Araneidae-214 **G, H** male holotype **A** epigyne, ventral view **B** ibid., posterior view **C** ibid., lateral view **D** vulva, anterior view **E** habitus, dorsal view **F** ibid., ventral view **G** ibid., dorsal view **H** ibid., lateral view. Scale bars: 0.1 mm (**A–D**); 1 mm (**E–H**). Abbreviations: CD copulatory duct, CO copulatory opening, FD fertilization duct, Sc scape, Sp spermatheca.

**Figure 10.**
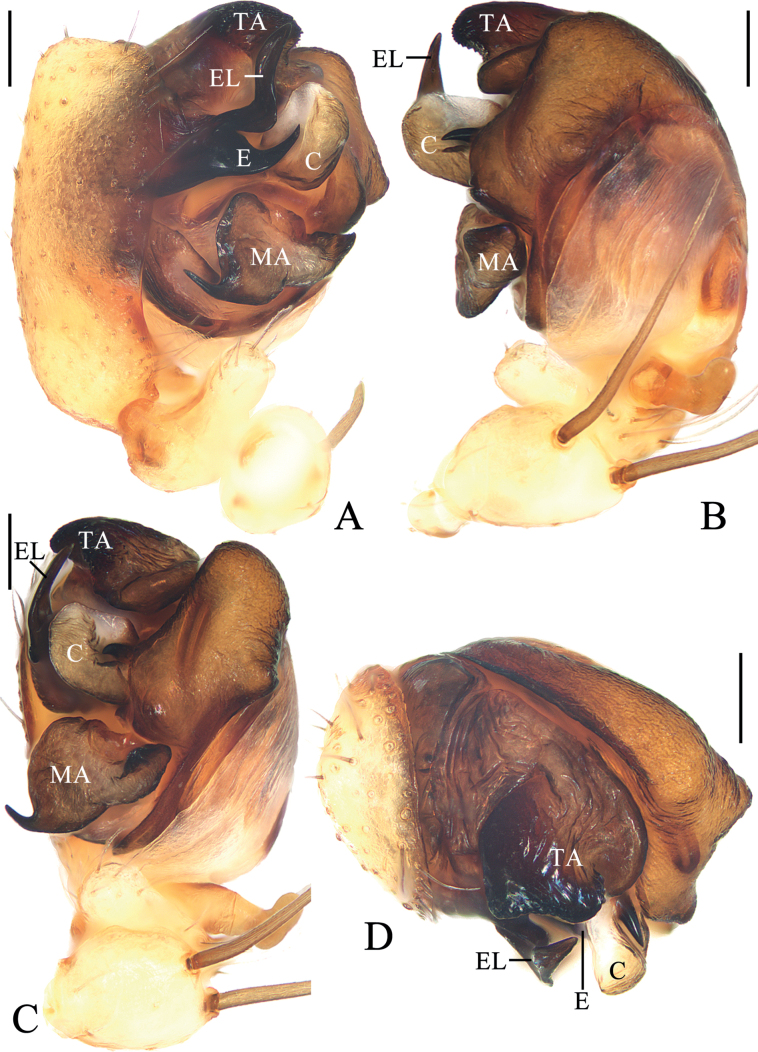
*Araneuswanghuai* sp. nov. male holotype **A** pedipalp, prolateral view **B** ibid., retrolateral view **C** ibid., ventral view **D** ibid., apical view. Scale bars: 0.1 mm. Abbreviations: C conductor, E embolus, EL embolic lamella, MA median apophysis, ST subterminal apophysis, TA terminal apophysis.

Figs 9 , 10 , 16A–D , 17

##### Type material.

***Holotype*** ♂ (TRU-Araneidae-213), China: Guizhou Province, Tongren City, Yinjiang Tujia and Miao Autonomous County, Ziwei Township, Dayuanzhi Village, Huguosi (27°54.72'N, 108°28.62'E, ca 1500 m), 9.V.2021, X.Q. Mi et al. leg. ***Paratypes***: 2♂2♀ (TRU-Araneidae-214–217), same data as for holotype; 1♂ (TRU-Araneidae-218), same locality as holotype, 24.IV.2020, X.Q. Mi & C. Wang leg.; 9♀ (TRU-Araneidae-219–227), Mianxüling (27°54.89'N, 108°40.17'E, ca 1790 m), 14.VI.2019, C. Wang & J.H. Gan leg.; 1♀ (TRU-Araneidae-228), Mianxüling (27°54.89'N, 108°40.17'E, ca 1790 m), 23.VII.2021, X.Q. Mi et al. leg.; 3♂2♀ (TRU-Araneidae-229–233), Mianxüling (27°54.83'N, 108°40.03'E, ca 2000 m), 9.V.2020, X.Q. Mi et al. leg.; 1♂ (TRU-Araneidae-234), Muhuang Township, Jinchang Village, Maxi’ao (28°1.37'N, 108°45.00'E, ca 1340 m), 26.IV.2020, X.Q. Mi & C. Wang leg.; 2♂ (TRU-Araneidae-235–236), same locality, 10.V.2020, X.Q. Mi et al. leg.

##### Other material examined.

1♂ (TRU-Araneidae-237), China: Guizhou Province, Qiandongnan Miao and Dong Autonomous Prefecture, Leishan County, Danjiang Township, Xiannütang, Leigongshan National Nature Reserve (26°22.38'N, 108°11.87'E, ca 1550 m), 29.IV.2018, X.Q. Mi et al. leg.; 1♀ (TRU-Araneidae-238), Danjiang Township, Leigongshan National Nature Reserve (26°22.86'N, 108°11.79'E, ca 1790 m), 30.IV.2018, X.Q. Mi et al. leg.

##### Comparative material.

*Araneusalbabdominalis* Zhu, Zhang, Zhang & Chen, 2005, 1♂1♀ (TRU-Araneidae-239–240), Huguosi (27°54.54'N, 108°46.57'E, ca 1660 m), 9.V.2020, X.Q. Mi et al. leg.

##### Diagnosis.

The new species resembles *A.albabdominalis* Zhu, Zhang, Zhang & Chen, 2005 in genital structures, but differs in: 1) distal embolic lamella slightly clockwise curved in prolateral view (Fig. 10A) vs anti-clockwise curved (Zhu et al. 2005: fig. 3D); 2) embolus curved distally (Fig. 10A) vs almost straight (Zhu et al. 2005: fig. 3D); 3) copulatory openings located at lateral surface (Fig. 9C) vs at ventral surface (Zhu et al. 2005: fig. 3B); 4) scape with nearly parallel sides (Fig. 9A) vs twisted into an S-shape (Zhu et al. 2005: fig. 3B); 5) female abdomen with a pair of lateral humps (Fig. 9E) vs lacking (Zhu et al. 2005: fig. 3A); 6) dorsal abdomen with pale patches (Fig. 9E, G) vs unicolor (Zhu et al. 2005: fig. 3A); and 7) yellow in life vs pale green in life.

##### Description.

**Male** (holotype, Figs 9G, H, 10, 16A–D). Total length 3.15. Carapace 1.75 long, 1.40 wide. Abdomen 2.00 long, 1.50 wide. Clypeus 0.10 high. Eye sizes and interdistances: AME 0.08, ALE 0.08, PME 0.10, PLE 0.08, AME–AME 0.13, AME–ALE 0.13, PME–PME 0.10, PME–PLE 0.18, MOA length 0.28, anterior width 0.25, posterior width 0.28. Leg measurements: I 6.20 (1.95, 2.10, 1.55, 0.60), II 5.95 (1.85, 2.00, 1.50, 0.60), III 3.30 (1.10, 1.00, 0.75, 0.45), IV 4.55 (1.50, 1.45, 1.10, 0.50). Carapace pear-shaped, yellow with yellowish brown bilateral sides, cervical groove slightly obvious, fovea longitudinal. Chelicerae yellow, four promarginal teeth and three retromarginal teeth. Endites square, with tooth-like process laterally, labium triangular, both yellow with pale tip. Sternum cordiform, yellow with dark setae. Legs yellow to yellowish brown, without annulus, tibia I with 12 macrosetae, tibia II with 12 macrosetae, tibia III with ten macrosetae, tibia IV with ten macrosetae. Abdomen oval, ~ 1.33× longer than wide, with a pair of lateromarginal humps on anterior surface, dorsum grayish yellow with a pair of longitudinal, white stripes anteriorly, and a whitish yellow transverse band medially, and four pairs of dark spots posterolaterally; venter grayish yellow. Spinnerets grayish yellow.

***Pedipalp*** (Fig. 10): with basal femoral protrusion; patella with two bristles; median apophysis large, triangular with two point tips; embolus stout at base, curved medially and tapered to a pointed tip, with a long, sclerotized embolic lamella; conductor weakly sclerotized, with a spur at base; terminal apophysis large, heavily sclerotized, with dozens of denticles.

**Female** (paratype TRU-Araneidae-214, Fig. 9A–F). Total length 4.40. Carapace 1.85 long, 1.50 wide. Abdomen 3.10 long, 3.25 wide. Clypeus 0.08 high. Eye sizes and interdistances: AME 0.08, ALE 0.08, PME 0.13, PLE 0.08, AME–AME 0.15, AME–ALE 0.18, PME–PME 0.13, PME–PLE 0.25, MOA length 0.33, anterior width 0.28, posterior width 0.30. Leg measurements: I 5.85 (1.80, 2.10, 1.40, 0.55), II 5.55 (1.75, 1.95, 1.30, 0.55), III 3.45 (1.15, 1.15, 0.70, 0.45), IV 4.95 (1.70, 1.65, 1.10, 0.50). Habitus similar to that of male but the abdomen slightly wider than long, and dorsal abdomen with a large triangular pale patch.

***Epigyne*** (Fig. 9A–D): scape with nearly parallel sides, distal end spoon shaped; copulatory openings elliptical, on lateral surface; copulatory ducts expanded, almost straight, longer than the spermatheca; spermathecae oval, nearly touching.

##### Variation.

Total length: ♂♂ 3.15–3.30 (*n* = 11); ♀♀ 3.55–4.40 (*n* = 15). Dark spots on abdomen sometimes inconspicuous.

##### Distribution.

Guizhou (Yinjiang, Leishan).

##### Comments.

The female abdomen wide oval with a pair of anterior lateral humps, the long, ridged scape, and the male pedipalp with a wide terminal apophysis indicate that the new species belongs to the *A.diadenmatus* group.

##### Etymology.

The species is named after Mr. Hua Wang (Fanjingshan National Nature Reserve Administration Bureau), who assisted greatly in field trips; noun in genitive case.

#### ﻿ Araneus yangchuandongi sp. nov.

Taxon classificationAnimaliaAraneaeAraneidae

A0CF9F9D-35F4-50CF-A1D5-F7B6EC9406CC

https://zoobank.org/9761C52A-00D2-4C9A-8F36-78E4230DC4AC

**Figure 11.**
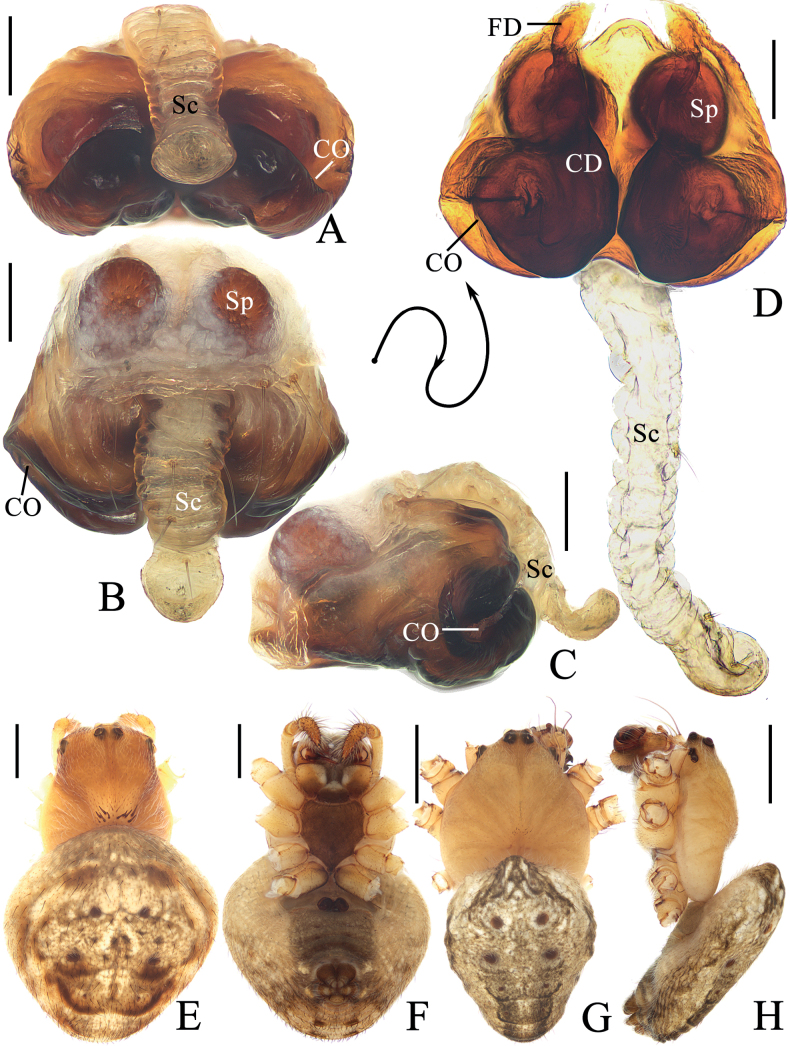
*Araneusyangchuandongi* sp. nov. **A–F** female paratype TRU-Araneidae-242 **G, H** male holotype **A** epigyne, ventral view **B** ibid., anterior view **C** ibid., lateral view **D** vulva, posterior view **E** habitus, dorsal view **F** ibid., ventral view **G** ibid., dorsal view **H** ibid., lateral view. Scale bars: 0.1 mm (**A–D**); 1 mm (**E–H**). Abbreviations: CD copulatory duct, CO copulatory opening, FD fertilization duct, Sc scape, Sp spermatheca.

**Figure 12.**
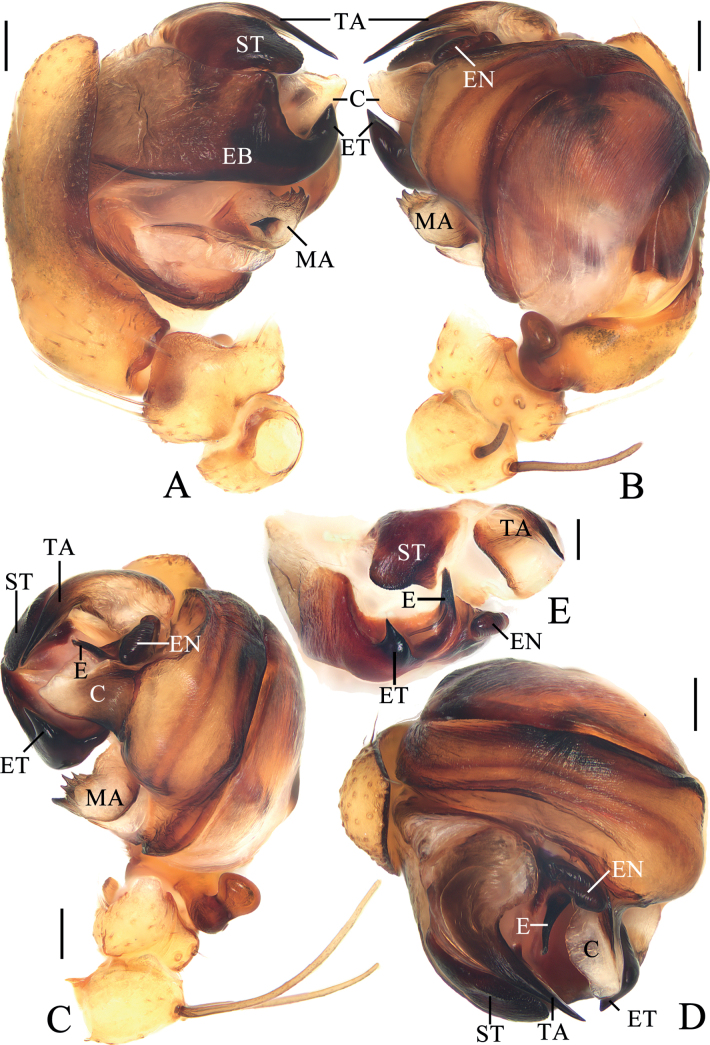
*Araneusyangchuandongi* sp. nov. male holotype **A** pedipalp, prolateral view **B** ibid., retrolateral view **C** ibid., ventral view **D** ibid., apical view **E** part of expanded bulb. Scale bars: 0.1 mm. Abbreviations: C conductor, E embolus, EB embolic base, EN embolic node, ET embolic tooth, MA median apophysis, ST subterminal apophysis, TA terminal apophysis.

Figs 11 , 12 , 16E–H , 17

##### Type material.

***Holotype*** ♂ (TRU-Araneidae-241), China: Guizhou Province, Tongren City, Yinjiang Tujia and Miao Autonomous County, Ziwei Township, Dayuanzhi Village, Huguosi (27°54.72'N, 108°28.62'E, ca 1500 m), 24.IV.2020, X.Q. Mi & C. Wang leg. ***Paratypes***: 1♀ (TRU-Araneidae-242), same locality as for holotype, 25.IV.2020, X.Q. Mi & C. Wang leg.; 2♀ (TRU-Araneidae-243–244), same locality as holotype, 8.V.2020, X.Q. Mi et al. leg.; 1♀ (TRU-Araneidae-245), same locality as holotype, 9.V.2020, X.Q. Mi et al. leg.; 1♀ (TRU-Araneidae-246), Dayuanzhi Village, Wanjuanshu (27°55.07'N, 108°37.67'E, ca 1200 m), 25.IV.2020, X.Q. Mi & C. Wang leg.; 1♀ (TRU-Araneidae-247), Huguosi (27°54.54'N, 108°46.57'E, ca 1660 m), 9.V.2020, X.Q. Mi et al. leg.; 1♂ (TRU-Araneidae-248), Songtao Miao Autonomous County, Wuluo Township, Taohuayuan Village (27°59.10'N, 108°46.15'E, ca 1230 m), 4.IV.2022, C. Wang & X. Chen leg.

##### Other material examined.

1♀ (TRU-Araneidae-249), China: Guizhou Province, Qiandongnan Miao and Dong Autonomous Prefecture, Leishan County, Danjiang Township, Xiannütang, Leigongshan National Nature Reserve (26°22.38'N, 108°11.87'E, ca 1550 m), 29.IV.2018, X.Q. Mi et al. leg.; 1♀ (TRU-Araneidae-250), same locality (26°22.86'N, 108°11.79'E, ca 1790 m), 30.IV.2018, X.Q. Mi et al. leg.; 1♀ (TRU-Araneidae-251), Danjiang Township, Xiangshuiyan, Leigongshan National Nature Reserve (26°21.73'N, 108°9.59'E, ca 1200 m), 1.V.2018, G.J. Tian & H. Liu leg.; 1♀ (TRU-Araneidae-252), Fangxiang Township, Queniao Village, Leigongshan National Nature Reserve (26°25.01'N, 108°13.78'E, ca 1150 m), 2.V.2018, X.Q. Mi et al. leg.; 1♀ (TRU-Araneidae-253), Tongren City, Shiqian County, Ganxi Township, Fuyan Village, Jiuchashu, Fodingshan National Nature Reserve (27°20.62'N, 108°3.56'E, ca 1410 m), 7.VI.2019, C. Wang et al. leg.

##### Diagnosis.

The new species resembles *A.colubrinus* Song & Zhu, 1992 in appearance, but differs in: 1) copulatory openings slit-shaped (Fig. 11A) vs deeply concaved (Fig. 3A); 2) scape with nearly parallel sides (Fig. 11A) vs twisted into an S-shape (Fig. 3A); 3) carapace with ten macrosetae anterior to fovea (Fig. 11E, G) vs lacking (Fig. 3E, H); 4) embolus tapered (Fig. 12C–E) vs threadlike (Fig. 4A–E); 5) terminal apophysis heavily sclerotized pointed at tip (Fig. 12A–E) vs membranous and lamellar (Fig. 4A–E); and 6) conductor not much longer than wide (Fig. 12A–D) vs ~ 3.6× longer than wide in retrolateral view (Fig. 4B).

##### Description.

**Male** (holotype, Figs 11G, H, 12, 16E–H). Total length 4.00. Carapace 2.15 long, 1.90 wide. Abdomen 2.70 long, 1.95 wide. Clypeus 0.08 high. Eye sizes and interdistances: AME 0.10, ALE 0.08, PME 0.13, PLE 0.08, AME–AME 0.18, AME–ALE 0.25, PME–PME 0.13, PME–PLE 0.35, MOA length 0.35, anterior width 0.38, posterior width 0.38. Leg measurements: I 8.40 (2.45, 3.05, 2.10, 0.80), II 7.60 (2.15, 2.60, 2.10, 0.75), III 4.60 (1.55, 1.55, 0.95, 0.55), IV 5.95 (1.85, 2.10, 1.40, 0.60). Carapace pear-shaped, yellow with pale setae, with ten macrosetae anterior to fovea, cervical groove slightly obvious. Chelicerae yellow, four promarginal teeth and three retromarginal teeth. Endites almost square, yellow, with tooth-like process laterally, labium triangular, grayish yellow. Sternum cordiform, yellow with inconspicuous dark patches and dark setae. Legs yellow to yellowish brown, without annulus, tibia I with 17 macrosetae, distally with constriction (see arrow in Fig. 16E), tibia II with 15 macrosetae, tibia III with nine macrosetae, tibia IV with ten macrosetae. Abdomen oval, pointed anteriorly and blunt posteriorly, ~ 1.38× longer than wide, covered with dense setae, dorsum whitish yellow with a triangular gray patch anteriorly and four pairs of arcuate gray patches posteriorly; venter grayish yellow. Spinnerets yellowish brown.

***Pedipalp*** (Fig. 12): with basal femoral protrusion; patella with two bristles; median apophysis bifurcated, with a large prolateral spur and four retrolateral teeth; embolus slender and straight, shorter than the conductor, bearing a tooth and a node, the embolic base about equal width to bulb diameter; conductor weakly sclerotized, not much longer than wide; terminal apophysis heavily sclerotized and pointed at tip, subterminal apophysis heavily sclerotized, somewhat flattened.

**Female** (paratype TRU-Araneidae-242, Fig. 11A–F). Total length 4.35. Carapace 2.10 long, 1.80 wide. Abdomen 3.30 long, 2.95 wide. Clypeus 0.05 high. Eye sizes and interdistances: AME 0.10, ALE 0.08, PME 0.13, PLE 0.08, AME–AME 0.15, AME–ALE 0.40, PME–PME 0.13, PME–PLE 0.45, MOA length 0.33, anterior width 0.33, posterior width 0.38. Leg measurements: I 6.75 (2.00, 2.50, 1.50, 0.75), II 5.60 (1.65, 2.00, 1.25, 0.70), III 3.70 (1.20, 1.25, 0.75, 0.50), IV 5.10 (1.55, 1.85, 1.15, 0.55). Habitus similar to that of male but the macrosetae anterior to fovea stronger, and anterior abdomen a little blunt.

***Epigyne*** (Fig. 11A–D): scape with nearly parallel sides, distal end spoon shaped; copulatory openings slit-like, on the ventral surface; copulatory ducts longer than spermatheca, curved to an S-shape; spermathecae round, nearly touching.

##### Variation.

Total length: ♂♂ 3.90–4.00 (*n* = 2); ♀♀ 4.10–6.20 (*n* = 12).

##### Distribution.

Guizhou (Yinjiang, Songtao, Leishan, Shiqian).

##### Comments.

The oval abdomen and the long, ridged, distally spoon-shaped scape indicate that the new species belongs to the *A.sturmi* group. The somatic morphology and genitalia indicate that the new species is most similar to *A.colubrinus*, *A.conexus*, and *A.zhoui*.

##### Etymology.

The species is named after Mr. Chuandong Yang (Fanjingshan National Nature Reserve Administration Bureau), who has been committed to biodiversity conservation of Fanjingshan National Nature Reserve for the past 40 years; noun in genitive case.

#### ﻿ Araneus yuboi sp. nov.

Taxon classificationAnimaliaAraneaeAraneidae

825E3301-41D8-5635-8153-C7B240ECE96B

https://zoobank.org/9D2757C4-7BB8-462C-B893-EAEB9F0A80A4

**Figure 13.**
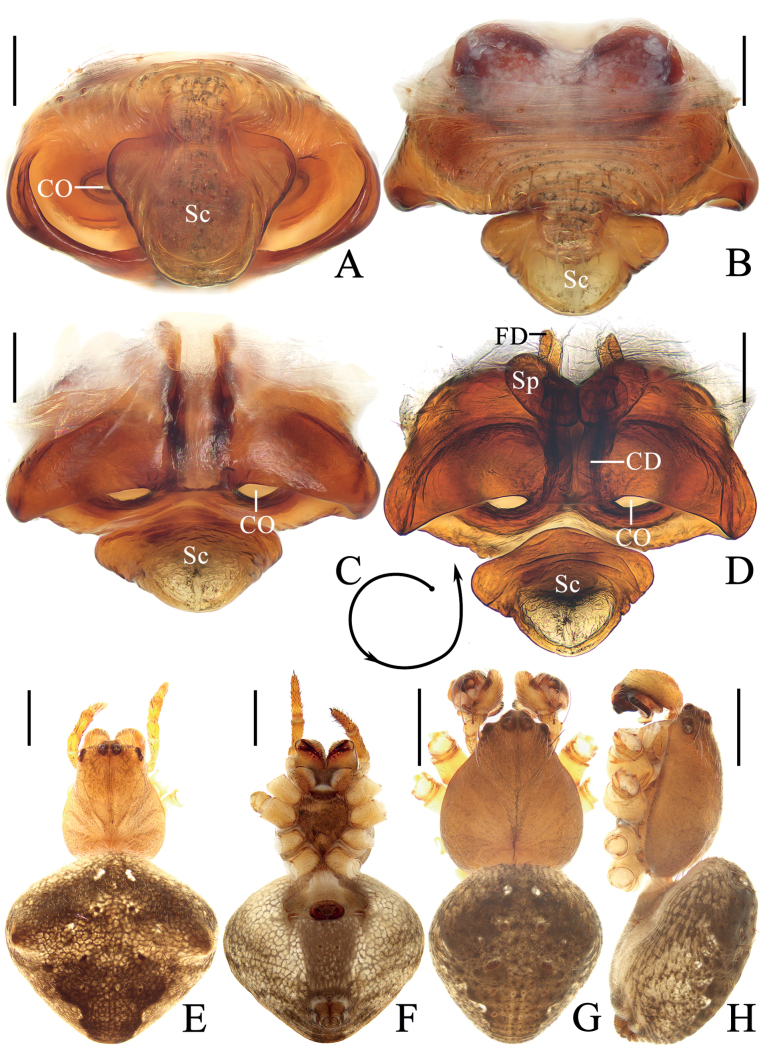
*Araneusyuboi* sp. nov. **A–F** female paratype TRU-Araneidae-255 **G, H** male holotype **A** epigyne, ventral view **B** ibid., anterior view **C** ibid., posterior view **D** vulva, posterior view **E** habitus, dorsal view **F** ibid., ventral view **G** ibid., dorsal view **H** ibid., lateral view. Scale bars: 0.1 mm (**A–D**); 1 mm (**E–H**). Abbreviations: CD copulatory duct, CO copulatory opening, FD fertilization duct, Sc scape, Sp spermatheca.

**Figure 14.**
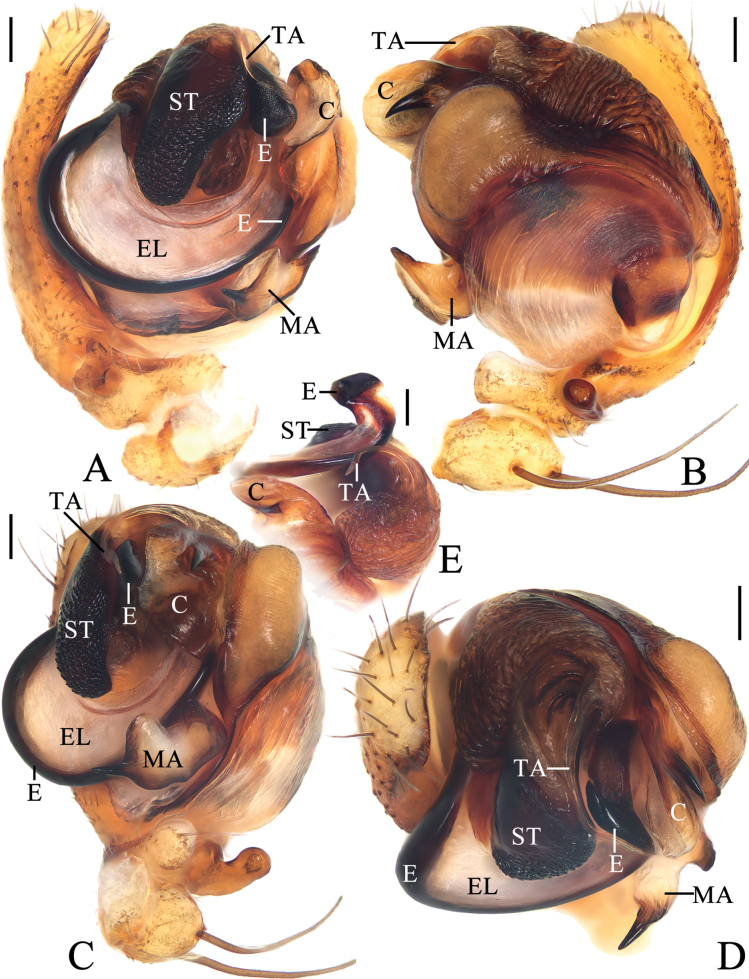
*Araneusyuboi* sp. nov. male holotype **A** pedipalp, prolateral view **B** ibid., retrolateral view **C** ibid., ventral view **D** ibid., apical view **E** part of expanded bulb. Scale bars: 0.1 mm. Abbreviations: C conductor, E embolus, EL embolic lamella, MA median apophysis, ST subterminal apophysis, TA terminal apophysis.

Figs 13 , 14 , 16I–L , 17

##### Type material.

***Holotype*** ♂ (TRU-Araneidae-254), China: Guizhou Province, Tongren City, Yinjiang Tujia and Miao Autonomous County, Ziwei Township, Dayuanzhi Village, Mianxüling (27°54.89'N, 108°40.17'E, ca 1790 m), 14.VI.2019, C. Wang & J.H. Gan leg; ***Paratypes***: 4♂4♀ (TRU-Araneidae-255–262), same data as for holotype; 2♂ (TRU-Araneidae-263–264), same locality (27°54.83'N, 108°40.03'E, ca 2000 m), 9.V.2020, X.Q. Mi et al. leg.; 3♀ (TRU-Araneidae-265–267), same locality (27°54.89'N, 108°40.17'E, ca 1790 m), 23.VII.2021, X.Q. Mi et al. leg.

##### Diagnosis.

The new species resembles *A.bimaculicollis* Hu, 2001 in appearance, but differs in: 1) scape with nearly parallel sides (Fig. 13A) vs twisted into an S-shape (Hu 2001: fig. 283.2); 2) scape with trapeziform tip in ventral view (Fig. 13A) vs spoon shaped tip (Hu 2001: fig. 283.2); 3) copulatory openings located on the ventral surface (Fig. 13A) vs on the ventral to posterior surface (Hu 2001: fig. 283.2); 4) spermathecae elliptical (Fig. 13D) vs C-shaped (Hu 2001: fig. 284.3); 5) conductor with a basal spur (Fig. 14B–D) vs lacking (Hu 2001: fig. 284.1, 2); and 6) male tibia I not expanded medially (Fig. 16I) vs expanded (Hu 2001: fig. 284.4).

##### Description.

**Male** (holotype, Figs 13G, H, 14, 16I–L). Total length 4.25. Carapace 2.20 long, 1.80 wide. Abdomen 2.50 long, 2.20 wide. Clypeus 0.08 high. Eye sizes and interdistances: AME 0.13, ALE 0.10, PME 0.13, PLE 0.10, AME–AME 0.15, AME–ALE 0.15, PME–PME 0.13, PME–PLE 0.28, MOA length 0.35, anterior width 0.38, posterior width 0.38. Leg measurements: I 9.05 (3.00, 3.20, 2.00, 0.85), II 8.05 (2.60, 2.80, 1.85, 0.80), III 4.85 (1.60, 1.70, 0.95, 0.60), IV 6.15 (2.00, 2.05, 1.45, 0.65). Carapace pear-shaped, yellowish brown with pale setae, cervical groove slightly obvious, fovea longitudinal. Chelicerae yellow, four promarginal teeth and three retromarginal teeth. Endites square, yellow, with tooth-like process laterally, labium triangular, grayish yellow, both with pale tip. Sternum cordiform, grayish yellow with dark setae. Legs yellow with brown annuli, tibia I with 13 macrosetae, tibia II with 12 macrosetae, tibia III with eight macrosetae, tibia IV with eight macrosetae. Abdomen wide oval, blunt anteriorly, ~ 1.13× longer than wide, covered with pale setae, dorsum grayish yellow with a pair of white spots anteriorly; venter grayish yellow. Spinnerets yellowish brown.

**Figure 15.**
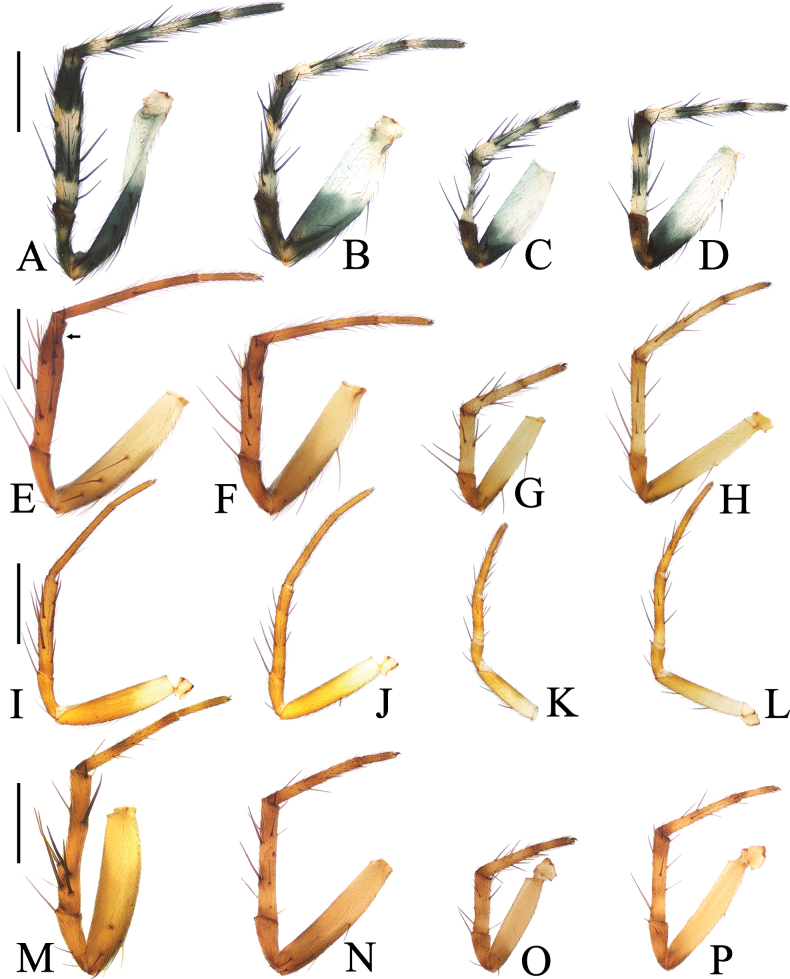
Legs of *Araneus* spp., male, prolateral view **A–D***A.chenjingi* sp. nov., holotype **E–H***Araneuscolubrinus* Song & Zhu, 1992 TRU-Araneidae-182 **I–L***A.lihaiboi* sp. nov., holotype **M–P***A.shii* sp. nov., holotype **A, E, I, M** legs I **B, F, J, N** legs II **C, G, K, O** legs III **D, H, L, P** legs IV. Scale bars: 1 mm.

**Figure 16.**
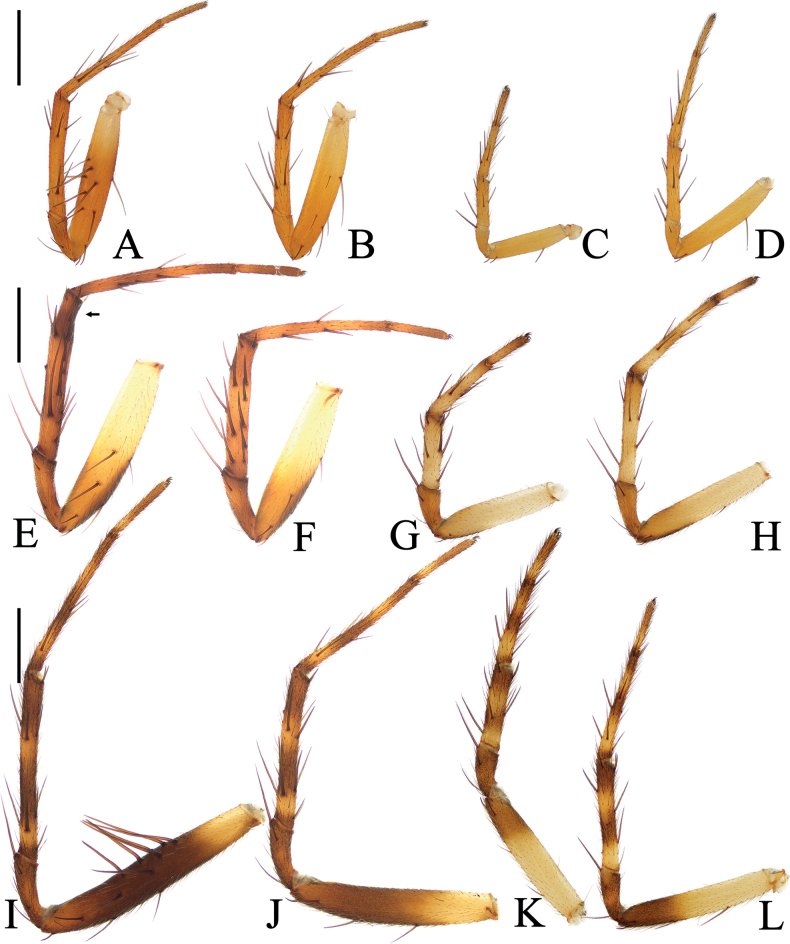
Legs of *Araneus* spp. male holotypes, prolateral view **A–D***A.wanghuai* sp. nov. **E–H***A.yangchuandongi* sp. nov. **I–L***A.yuboi* sp. nov. **A, E, I** legs I **B, F, J** legs II **C, G, K** legs III **D, H, L** legs IV. Scale bars: 1 mm.

***Pedipalp*** (Fig. 14): with basal femoral protrusion; patella with two bristles; median apophysis large, with a pointed tip and two fin-shaped protuberances; embolus extremely long, twisted into a U-shape, distal end enlarged and heavily sclerotized, with wide membranous embolic lamella; conductor weakly sclerotized, with a spur at base; terminal apophysis weakly sclerotized, digitiform; subterminal apophysis prominent, heavily sclerotized with dozens of denticles.

**Female** (paratype TRU-Araneidae-255, Fig. 13A–F). Total length 5.55. Carapace 2.45 long, 1.90 wide. Abdomen 3.50 long, 3.85 wide. Clypeus 0.05 high. Eye sizes and interdistances: AME 0.13, ALE 0.10, PME 0.13, PLE 0.10, AME–AME 0.13, AME–ALE 0.30, PME–PME 0.15, PME–PLE 0.38, MOA length 0.35, anterior width 0.38, posterior width 0.38. Leg measurements: I 8.65 (2.70, 3.15, 1.95, 0.85), II 7.65 (2.35, 2.80, 1.70, 0.80), III 4.70 (1.55, 1.55, 0.95, 0.60), IV 6.60 (2.15, 2.30, 1.50, 0.65). Habitus similar to that of male but abdomen slightly wider than long and with a pair of anterolateral humps.

***Epigyne*** (Fig. 13A–D): ~ 1.6× wider than long; scape almost straight, distal end trapeziform; copulatory openings widened and deeply concaved, on the ventral surface; copulatory ducts long, coiled about 360°; spermathecae elliptical, touching each other.

**Figure 17.**
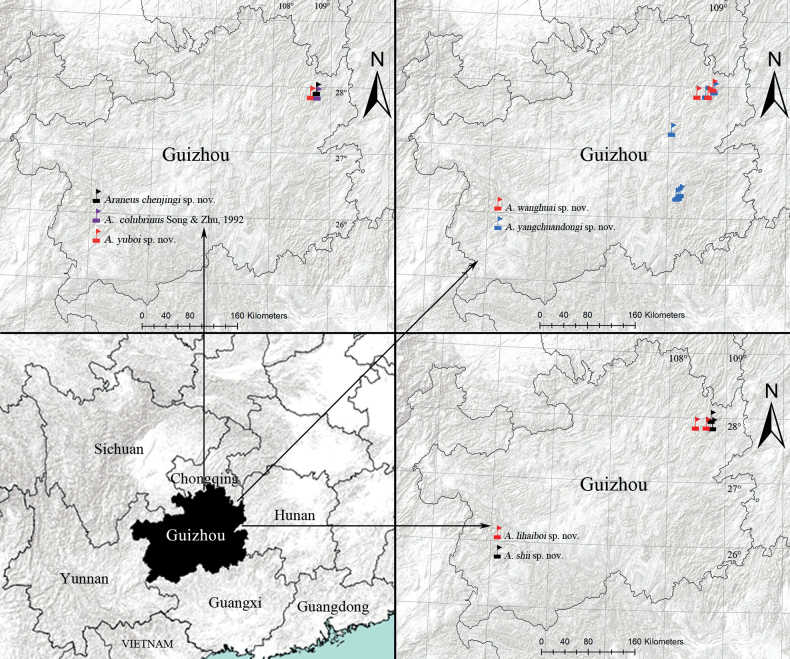
Distribution map of the species.

##### Variation.

Total length: ♂♂ 3.30–4.25 (*n* = 7); ♀♀ 4.30–5.55 (*n* = 7).

##### Distribution.

Known only from type locality.

##### Comments.

The female abdomen wide oval with a pair of anterolateral humps, the long, ridged scape indicate that the new species belongs to the *A.diadenmatus* group.

##### Etymology.

The species is named after Mr. Bo Yu (Fanjingshan National Nature Reserve Administration Bureau), who accompanied us on field collections; noun in genitive case.

## Supplementary Material

XML Treatment for Araneus

XML Treatment for Araneus chenjingi

XML Treatment for Araneus colubrinus

XML Treatment for Araneus lihaiboi

XML Treatment for Araneus shii

XML Treatment for Araneus wanghuai

XML Treatment for Araneus yangchuandongi

XML Treatment for Araneus yuboi

## ﻿Additional information

### Author ORCIDs

Xiaoqi Mi https://orcid.org/0000-0003-1744-3855

### Data availability

All of the data that support the findings of this study are available in the main text.
